# Supplementary material for: The Impact of COVID-19 on Hyperhidrosis Patients in the Mental Health and Quality of Life: A Web-Based Surveillance Study
Source: J Clin Med. 2022 Jun 21;11(13):3576. doi: 10.3390/jcm11133576 (PMC9267178; doi:10.3390/jcm11133576)

Supplementary Table S1. Online questionnaire for the study

COVID-19 related questionnaire					
**Question 1. After the COVID-19 pandemic, how would you rate the quality of life considering your hyperhidrosis symptoms and severity?**					
**NO**	**Answer**				
1	**Working environment**				
	Very good	Good	Same	Poor	Very poor
2	**The use of public spaces**				
	Very good	Good	Same	Poor	Very poor
3	**Academic activity (exam, interview, study)**				
	Very good	Good	Same	Poor	Very poor
4	**Digital life (the use of smartphone, devices and computers)**				
	Very good	Good	Same	Poor	Very poor
5	**Hobbies and leisure activity**				
	Very good	Good	Same	Poor	Very poor
6	**Intimate personal contacts (holding hands, hug, kiss, sexual intercourse)**				
	Very good	Good	Same	Poor	Very poor
7	**Public speaking or presentation**				
	Very good	Good	Same	Poor	Very poor
8	**Dressing (clothes, gloves, shoes, boots)**				
	Very good	Good	Same	Poor	Very poor
9	**Interpersonal relationship**				
	Very good	Good	Same	Poor	Very poor

Question 2. How would you rate the following problems related to hyperhidrosis during the COVID-19 pandemic?		
**NO**	**Answer**	
10	Did you experience the change of symptoms during the pandemic?	① Much worsened<br>② Worsened<br>③ Same as usual<br>④ Better<br>⑤ Much Better
11	Was it difficult to have appropriate management for hyperhidrosis during the pandemic?	① Yes<br>② Same as the pre-pandemic period<br>③ No
12	If you had problems in treatments during the pandemic, what were specific problems?	① No difficulty<br>② Visit to outpatient clinics<br>③ Worsened symptoms<br>④ Communication with physicians<br>⑤ Side effects<br>⑥ Insufficient information<br>⑦ Inaccurate information<br>⑧ Others

13	What kinds of treatment you received during the pandemic?	① No treatment<br>② Self-medication/Pharmacy<br>③ Herbal medicine<br>④ Health supplements<br>⑤ Surgery<br>⑥ Outpatient visit to dermatologists<br>⑦ Outpatient visit to thoracic surgeons<br>⑧ Mobile consultation<br>⑨ Leisure activity<br>⑩ Diet therapy<br>⑪ Others

Q. Over the last two weeks, how often have you been bothered by any of the following problems?

Over the last two weeks,		Not at all	Several days	More than half the days	Nearly everyday
14	Little interest or pleasure in doing things?	**0**	**1**	**2**	**3**
15	Feeling down, depressed, or hopeless?	**0**	**1**	**2**	**3**
16	Trouble falling or staying asleep, or sleeping too much?	**0**	**1**	**2**	**3**

17	Feeling tired or having little energy?	**0**	**1**	**2**	**3**
18	Poor appetite or overeating?	**0**	**1**	**2**	**3**
19	Feeling bad about yourself - or that you are a failure or have let yourself or your family down?	**0**	**1**	**2**	**3**
20	Trouble concentrating on things, such as reading the newspaper or watching television?	**0**	**1**	**2**	**3**
21	Moving or speaking so slowly that other people could have noticed? Or the opposite - being so fidgety or restless that you have been moving around a lot more than usual?	**0**	**1**	**2**	**3**
22	Thoughts that you would be better off dead, or of hurting yourself in some way?	**0**	**1**	**2**	**3**
**Total score (0-27)**					

Supplementary Table S2. Age/gender/disease duration-matched participants' demographic, hyperhidrosis characteristics, and PHQ-9 score

	**Male**<br>n=84	**Female**<br>n=84	***P value***
Demographics			
Age	39.00 [33.75, 44.25]	39.00 [33.75, 44.25]	0.93
Age group			0.957
20-29	8 (9.5)	11 (13.1)	
30-39	35 (41.7)	33 (39.3)	
40-49	32 (38.1)	31 (36.9)	
50-59	8 (9.5)	8 (9.5)	
60+	1 (1.2)	1 (1.2)	
Marital status			*0.758*
married	42 (50.0)	39 (46.4)	
unmarried	42 (50.0)	45 (53.6)	
Residential area			
Small town	28 (33.3)	25 (29.8)	*0.740*
Urban/Metropolitan area	56 (66.7)	59 (70.2)	
Depression related			
PHQ-9 mean (SD)	4.05 (5.24)	6.96 (6.77)	***0.002***
PHQ-9 median (IQR)	2.00 [0.00, 6.00]	4.00 [2.00, 10.25]	***0.001***
Mild Depression¶	26 (31.0)	41 (48.8)	***0.027***
Moderate Depression⁑	14 (16.7)	26 (31.0)	***0.045***
Hyperhidrosis related			
Duration	30.00 [20.00, 32.50]	29.00 [20.75, 35.00]	0.620
Axilla	38 (45.2)	51 (60.7)	*0.063*
Palmar	59 (70.2)	63 (75.0)	*0.604*
Plantar	60 (71.4)	64 (76.2)	*0.599*
Facial	32 (38.1)	29 (34.5)	*0.748*
Multiple	67 (79.8)	75 (89.3)	*0.134*
Comorbidity⁂			

Gastrointestinal diseases	27 (32.1)	37 (44.0)	*0.153*
Obesity	27 (32.1)	23 (27.4)	*0.613*
Anxiety	13 (15.5)	19 (22.6)	*0.326*
Depression	13 (15.5)	22 (26.2)	*0.128*
Hypertension	14 (16.7)	4 (4.8)	***0.023***
Diabetes mellitus	5 (6.0)	2 (2.4)	*0.443*
Neurologic disorder	8 (9.5)	8 (9.5)	*1*

BMI, body-mass index; SD, standard deviation; IQR, interquartile range; PHQ-9, patient health questionnaire-9

¶ Patients with PHQ-9 score 5 or above

⁑ Patients with PHQ-9 score 10 or above

⁂This is based on the patients' self-report not previous medical records

Supplementary Table S3. Age/gender/disease duration matched participants' demographic, hyperhidrosis characteristics and PHQ-9 score

	Male<br>n=84	Female<br>n=84	*P value*
Quality of life problems			
Use of public spaces, (%)	34 (40.5)	49 (58.3)	***0.030***
Working environments (%)	11 (13.1)	21 (25.0)	*0.076*
Interpersonal relationship (%)	32 (38.1)	46 (54.8)	***0.044***
Public speaking or presentation (%)	43 (51.2)	58 (69.0)	***0.027***
Digital life¶ (%)	23 (27.4)	29 (34.5)	*0.404*
Intimate personal contacts (%)	49 (58.3)	57 (67.9)	*0.263*
Dressing (%)	37 (44.0)	47 (56.0)	*0.165*
Hobbies and leisure activity (%)	25 (29.8)	35 (41.7)	*0.147*
Academic activity (%)	35 (41.7)	44 (52.4)	*0.216*
Change in symptoms			*0.576*
Improved	3 (3.6)	2 (2.4)	
Same as usual	74 (88.1)	70 (83.3)	
Worsened	7 (8.3)	12 (14.3)	
Problems in treatments during the pandemic			
No problem	62 (73.8)	55 (65.5)	*0.314*
Information related issues	11 (13.1)	13 (15.5)	*0.826*
Inaccurate information	5 (6.0)	4 (4.8)	*1*
Insufficient information	8 (9.5)	12 (14.3)	*0.476*
Visit to outpatient clinics	11 (13.1)	10 (11.9)	*1*
Side effects	4 (4.8)	4 (4.8)	*1*
Communication with physicians	3 (3.6)	1 (1.2)	*0.622*
Treatments received during the pandemic			
No treatment	47 (56.0)	35 (41.7)	*0.089*

Self-medication⁂	17 (20.2)	30 (35.7)	***0.039***
OPD visit to thoracic surgeons	8 (9.5)	6 (7.1)	*0.781*
OPD visit to dermatologist	2 (2.4)	4 (4.8)	*0.682*
Surgery	3 (3.6)	0 (0.0)	*0.246*
Diet therapy	7 (8.3)	6 (7.1)	*1*
Leisure activity	6 (7.1)	5 (6.0)	*1*
Herbal medicine	1 (1.2)	1 (1.2)	*1*
Mobile consultation	0 (0.0)	1 (1.2)	*1*
Health supplement	4 (4.8)	6 (7.1)	*0.746*
Others	8 (9.5)	10 (11.9)	*0.804*

OPD, outpatient department

¶ It includes inconvenience related to the use of smartphone and electronic devices

⁂ Over the counter drugs, aesthetic products and the use of topical agents such as aluminum chloride

Supplementary Figure S1(a). The Spearman's rank correlation between PHQ-9 and age

- **PHQ-9 and Age**
    - Spearman's rank correlation coefficient -0.264
    - P value = 0.0000691

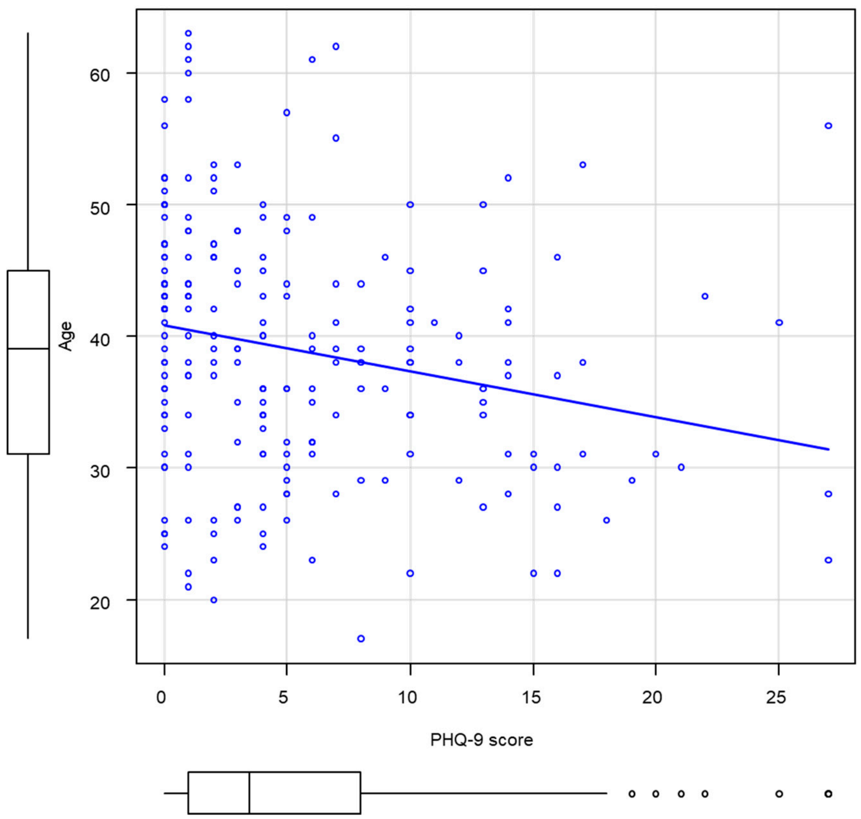

Supplementary Figure S1(b). The Spearman’s rank correlation between PHQ-9 and the hyperhidrosis duration

- **PHQ-9 and Hyperhidrosis duration**
    - Spearman's rank correlation coefficient -0.212
    - P value = 0.00149

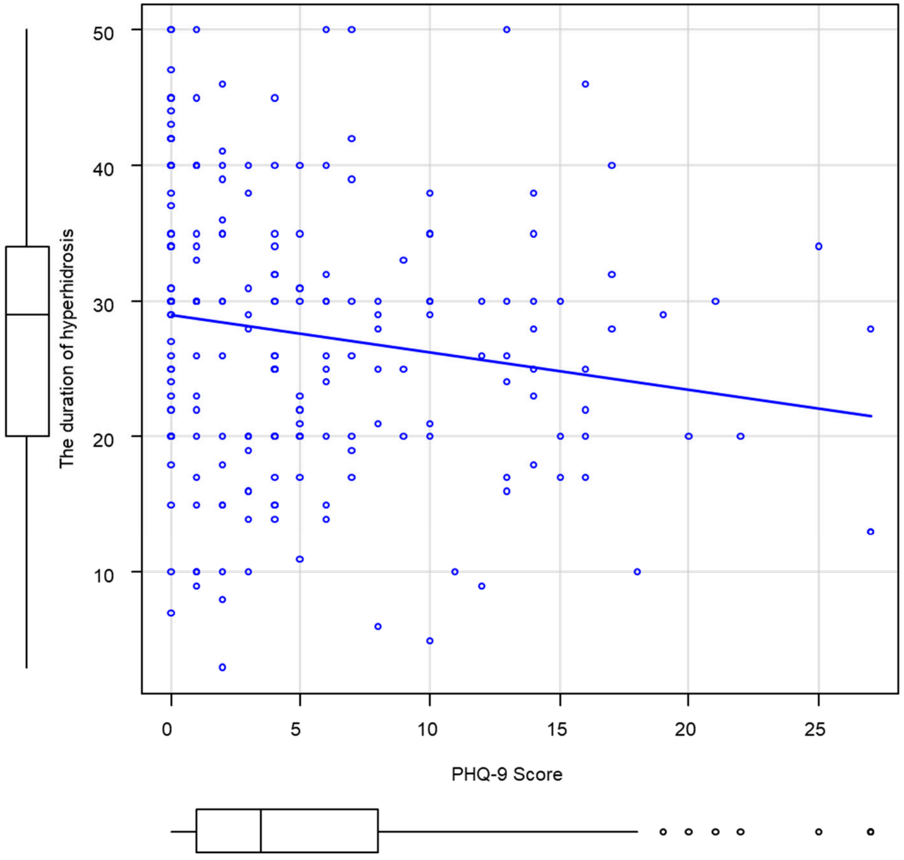

Supplementary Figure S1(c). The Spearman's rank correlation between PHQ-9 and body-mass index

- **PHQ-9 and BMI**
- Spearman's rank correlation coefficient -0.127
- P value = 0.0597

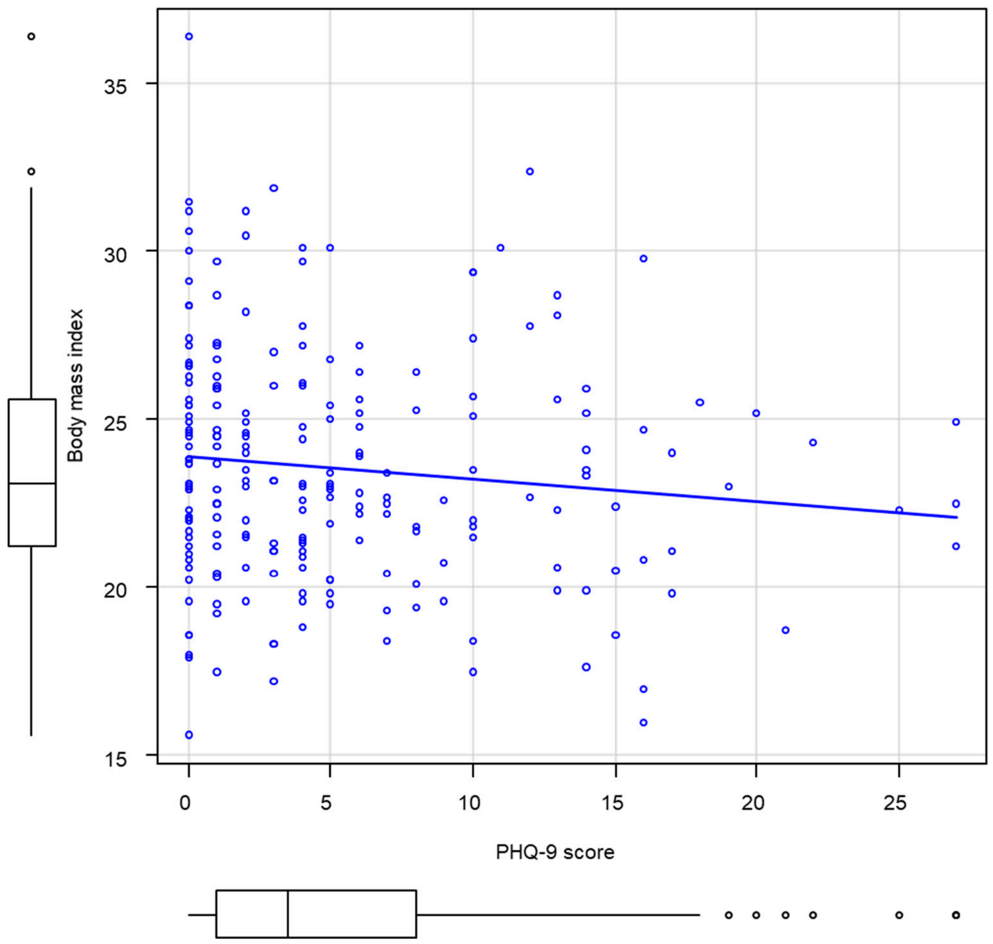

Supplement: Supplementary file 1 [file jcm-11-03576-s001.zip › jcm-1768857-supplementary.pdf]
